# Taurohyocholic acid acts as a potential predictor of the efficacy of tyrosine kinase inhibitors combined with programmed cell death-1 inhibitors in hepatocellular carcinoma

**DOI:** 10.3389/fphar.2024.1364924

**Published:** 2024-02-23

**Authors:** Yue Chen, Yutao Wang, Jin Lei, Bowen Chen, Xinfeng Zhang, Liangzheng Chang, Zhangli Hu, Yun Wang, Yinying Lu

**Affiliations:** ^1^ Department of Infectious Diseases, The Affiliated Hospital of Guizhou Medical University, Guiyang, China; ^2^ Senior Department of Hepatology, The Fifth Medical Center of PLA General Hospital, Beijing, China; ^3^ Peking University 302 Clinical Medical School, Beijing, China; ^4^ Institute of Molecular Medicine (IMM), Renji Hospital, State Key Laboratory of Oncogenes and Related Genes, Shanghai Jiao Tong University School of Medicine, Shanghai, China; ^5^ The PLA 307 Clinical College of Anhui Medical University, The Fifth Clinical Medical College of Anhui Medical University, Hefei, China; ^6^ Longhua Innovation Institute for Biotechnology, College of Life Sciences and Oceanography, Shenzhen University, Shenzhen, China

**Keywords:** hepatocellular carcinoma, tyrosine kinase inhibitor, programmed death-1 inhibitor, biomarkers, bile acid, treatment response

## Abstract

**Background and aims:** Tyrosine kinase inhibitors (TKIs) combined with programmed cell death protein-1 (PD-1) have significantly improved survival in patients with unresectable hepatocellular carcinoma (uHCC), but effective biomarkers to predict treatment efficacy are lacking. Peripheral blood bile acids (BAs) are associated with tumor response to therapy, but their roles in HCC remain unclear.

**Methods:** This retrospective study included HCC patients who received first-line TKIs combined with PD-1 inhibitors treatment (combination therapy) in our clinical center from November 2020 to June 2022. The aim of this study was to analyze the changes in plasma BA profiles before and after treatment in both the responding group (Res group) and the non-responding group (Non-Res group). We aimed to explore the potential role of BAs in predicting the response to combination therapy in HCC patients.

**Results:** Fifty-six patients with HCC who underwent combination therapy were included in this study, with 28 designated as responders (Res group) and 28 as non-responders (Non-Res group). There were differences in plasma BA concentrations between the two groups before systemic therapy. Plasma taurohyocholic acid (THCA) levels in the Res group were significantly lower than those in the Non-Res group. Patients with low levels of THCA exhibited superior median progression-free survival (7.6 vs. 4.9 months, *p* = 0.027) and median overall survival (23.7 vs. 11.6 months, *p* = 0.006) compared to those of patients with high levels of THCA.

**Conclusion:** Peripheral blood BA metabolism is significantly correlated with combination therapy response and survival in patients with HCC. Our findings emphasize the potential of plasma BAs as biomarkers for predicting combination therapy outcomes and offering novel therapeutic targets for modulating responses to systemic cancer therapy.

## 1 Introduction

Hepatocellular carcinoma (HCC) is currently the third most common cause of cancer-related deaths globally, and its incidence is on the rise (Devarbhavi et al., 2023). Systemic therapy is the first-line treatment option for advanced HCC, encompassing targeted therapy, such as tyrosine kinase inhibitors (TKIs), immune-checkpoint inhibitors (ICIs), including programmed cell death-1 (PD-1) inhibitors, and combined therapies (Xu et al., 2018). Study 117 found that lenvatinib plus the anti-PD-1 monoclonal antibody nivolumab resulted in an objective response rate of 66.7% and a clinical benefit rate of 70.8% in patients with advanced HCC (Kudo et al., 2020). LEAP-002 study showed that the lenvatinib combined with the pembrolizumab regimen had a trend of better benefit compared with lenvatinib monotherapy in the Asian subgroup (approximately 50% of Chinese patients), with a median PFS increase of 1.8 months, an ORR increase of 9.2%, and a median OS increase of approximately 4 months (Llovet et al., 2023). Another multicenter real-world study conducted by our clinical center and Peking Union Medical College Hospital showed that the median OS was 17.8 months, the median PFS was 6.9 months, and the ORR was 19.6% (Yang et al., 2023). Despite the effectiveness of these therapies for the treatment of HCC in terms of tumor response and patient survival, only a proportion of patients fully benefit from immunotherapy. Therefore, it is imperative to identify biomarkers that can predict outcomes of combination therapies to screen for patients with high potential long-term benefits and promote the development of personalized and precision medicine.

Dysregulation of bile acids (BAs) has been noted in various liver diseases, such as liver cirrhosis, nonalcoholic fatty liver disease, and HCC (Puri et al., 2018; Xie et al., 2021; Shen et al., 2022). In our previous study, we found significant changes in BA levels in patients with primary liver cancer compared with those with cirrhosis and healthy individuals, especially a significant increase in tauroursodeoxycholic acid (TUDCA) levels in plasma, which was negatively correlated with survival (Jia et al., 2020). Chen et al. (2011) found that the levels of glycocholic acid (GCA) and chenodeoxycholic acid glycine conjugate (GCDCA) were negatively correlated with the stage of HCC after analyzing the plasma BA components of patients with HCC at different stages, suggesting that the levels of these two components had the potential to predict the progression of HCC. In addition, several studies have suggested a role for BAs in treatment response. Patients who reacted to ICI had significantly greater amounts of ursodeoxycholic acid (UDCA) and ursocholic acid present in their feces compared to those who did not react (Lee et al., 2022). Secondary BAs have been demonstrated to regulate the build-up of CXCR6+ natural killer T cells in preclinical tumor models, inhibiting the progression of liver cancer, which is a potential mechanism underlying the role of BAs in responding to immunotherapy (Ma et al., 2018). Nevertheless, the relationship between plasma BAs and target-immune combination therapy in HCC remains unclear.

Furthermore, a similar phenomenon has been observed in patients with breast cancer, with a robust connection between plasma BA metabolite composition, host immune activation, and therapeutic response (Wu et al., 2022).

In this study, we examined the disparities in BA profiles between patients with HCC who exhibited clinical efficacy from combination therapy and those who did not. The aim of this study was to discover plasma biomarkers indicating target-immune combination therapy response and outcomes in patients with HCC, aiming to pinpoint subgroups that might benefit from combination therapies and maximize patient benefits.

## 2 Materials and methods

### 2.1 Patients

This retrospective study was approved by the Ethics Committee of the Fifth Medical Center of the PLA General Hospital (KY-2021-12-35-1). The research was carried out in compliance with the ethical standards outlined in the Declaration of Helsinki. Patients with hepatitis B virus (HBV)-related HCC who received TKIs combined with PD-1 inhibitors therapy (combination therapy) as a first-line treatment between November 2020 and June 2022 were considered for inclusion. The inclusion criteria were as follows: 1) histologically or radiologically diagnosed HCC; 2) unresectable HCC, i.e., not eligible for curative treatment; 3) age 18 years or older; 4) Child–Pugh score class A–B; 5) Eastern Cooperative Oncology Group (ECOG) performance status (PS) scores of 0–2; 6) availability of plasma samples obtained at the beginning of systemic therapy; and 7) availability of complete clinical and follow-up data. The exclusion criteria were as follows: 1) presence of other primary malignant tumors; 2) prior treatment with systemic drug therapy; and 3) irregular medication. Plasma samples were obtained from the Biobank of the Fifth Medical Center of PLA General Hospital. All informed consent was obtained from the subjects, and all samples were promptly frozen at −80°C until analysis.

### 2.2 Evaluation of treatment response

Treatment response was assessed using dynamic computed tomography or magnetic resonance imaging at the baseline and every 6–8 weeks post-treatment initiation, according to the Response Evaluation Criteria in Solid Tumors (RECIST) version 1.1. Patients achieving a complete response (CR), partial response (PR), or stable disease (SD) for a minimum of 6 months were classified as responders (Res), whereas individuals demonstrating stable disease for less than 6 months or progressing disease (PD) were categorized as non-responders (Non-Res). Overall survival (OS) was determined from the commencement of the combination therapy until death for any reason. Survivors were censored at the last follow-up. Progression-free survival (PFS) was defined as the time from the initial dose to the first radiologically confirmed tumor progression or all-cause mortality, whichever occurred first.

### 2.3 Measurement of plasma BAs

Plasma samples (50 μL) were added to 400 μL acetonitrile/methanol solvent and mixed by vibration for 20 min. Aliquots (250 μL) of the supernatant were then lyophilized. The samples were reconstituted prior to analysis with 40 μL acetonitrile/methanol. BA analysis was performed using ultra-high-performance liquid chromatography-tandem mass spectrometry (ACQUITY UPLC-Xevo TQ-S, Waters Corp, Milford, MA, United States) as previously described (Toledo et al., 2017), and MassLynx software version 4.1 (Waters Corp). Internal standards were added to the test samples to monitor analytical bias throughout sample processing and analysis. Quality control samples were inserted into the entire injection sequence at intervals to objectively evaluate the intra-batch repeatability of large-scale samples and to correct the inter-batch error. BAs were quantified in plasma samples using the quantification curves and mass spectra obtained from standard samples analyzed under the same conditions.

### 2.4 Statistical analysis

Continuous variables are presented as median (interquartile range), whilst categorical variables are represented as frequencies and percentages. Chi-squared and Fisher’s exact tests were utilized for the comparison of categorical variables, and the paired *t*-test, Mann–Whitney U test, and Wilcoxon signed-rank test were adopted for continuous variables. The area under the receiver operating characteristic curve (AUROC) was calculated to determine the optimal cut-off values for BA levels. The optimal cut-off was considered to be the value with the highest Youden’s index (sensitivity + specificity − 1). The PFS and OS were determined using the Kaplan–Meier methodology and analyzed using the log-rank test. Univariate Cox regression analysis was performed on clinical and BA data, and multivariate Cox regression analysis was performed on significant factors (*p* < 0.05) identified by the univariate analysis. All statistical analyses were conducted using R software version 4.3.1 (R Project for Statistical Computing, Vienna, Austria) and SPSS software version 26 (IBM Corp, Armonk, NY, United States). All statistical tests were conducted using a two-sided approach, and statistical significance was determined as *p* < 0.05.

## 3 Results

### 3.1 Patient characteristics

Between November 2020 and June 2022, the study included 56 patients who met the specified criteria. According to RECIST version 1.1, 17, 20, and 19 patients exhibited PR, SD, and PD, respectively; there were 28 patients each in the Res and Non-Res groups. Most patients were male (*n* = 49, 87.5%) and under 60 years old (*n* = 37, 66.1%). At the time of enrollment, 43 (76.8%) patients exhibited portal vein invasion by the tumor, whereas 24 (42.9%) exhibited extrahepatic metastasis. The majority of the patients (*n* = 52, 92.9%) were classified as stage C according to the Barcelona Clinic Liver Cancer staging system, and Child–Pugh score class A (*n* = 42, 75%). The group was dominated by Lenvatinib plus anti-PD-1 therapy (*n* = 40, 71%). No significant differences were observed in tumor number, tumor size, alpha-fetoprotein levels, portal hypertension, and albumin levels between the Res and Non-Res groups, suggesting that there were no established confounding variables causing discrimination between the groups before sample gathering. The detailed baseline characteristics of the patients are outlined in Table 1.

**TABLE 1:** Clinical characteristics of 56 patients with unresectable hepatocellular carcinoma receiving tyrosine kinase inhibitors combined with programmed cell death-1 inhibitors therapy.

Characteristics	Res (*n* = 28)	Non-Res (*n* = 28)	*p*-Value
Age (<60 years), n (%)	16 (57.1%)	21 (75%)	0.158
Sex (male), n (%)	24 (85.7%)	25 (89.3%)	1.000
Diabetes, n (%)	9 (32.1%)	8 (28.6%)	0.771
Hypertension, n (%)	15 (53.6%)	9 (32.1%)	0.105
Smoking history, n (%)	14 (50%)	14 (50%)	1.000
Drinking history, n (%)	9 (32.1%)	9 (32.1%)	1.000
BMI (<24), n (%)	16 (57.1%)	17 (60.7%)	0.786
Tumor size (≥5 cm), n (%)	16 (57.1%)	21 (75%)	0.158
Multiple tumors, n (%)	15 (53.6%)	13 (46.4%)	0.593
Portal vein invasion, n (%)	19 (67.9%)	24 (85.7%)	0.114
Extrahepatic metastasis, n (%)	14 (50%)	10 (35.7%)	0.280
AFP (≥400 ng/mL), n (%)	11 (39.3%)	10 (35.7%)	0.783
Portal hypertension, n (%)	17 (60.7%)	17 (60.7%)	1.000
Splenomegalia, n (%)	24 (85.7%)	23 (82.1%)	1.000
Cirrhosis, n (%)	24 (85.7%)	26 (92.9%)	0.666
Ascites, n (%)	13 (46.4%)	15 (53.6%)	0.593
ALT (>40 U/L)	9 (32.1%)	13 (46.4%)	0.274
AST (>40 U/L)	16 (57.1%)	21 (75%)	0.158
Total bilirubin (μmol/L)	14.2 (10.975, 21.275)	16.3 (12.675, 22.4)	0.523
Albumin (g/L)	37.5 (34, 39.5)	36 (33, 38.25)	0.355
INR	1.115 (1.0525, 1.2225)	1.165 (1.0375, 1.2)	0.605
BCLC stage, n (%)			0.604
B	3 (10.7%)	1 (3.6%)	
C	25 (89.3%)	27 (96.4%)	
Child–Pugh class, n (%)			0.217
A	23 (82.1%)	19 (67.9%)	
B	5 (17.9%)	9 (32.1%)	
Treatment modality			0.554
Lenvatinib + PD-1 inhibitors	21 (37.5%)	19 (33.9%)	
Sorafenib + PD-1 inhibitors	7 (12.5%)	9 (16.1%)	

Notes: Data are presented as median with interquartile range (IQR), or n (%).

Abbreviations: BMI, body mass index; AFP, alpha-fetoprotein; AL(S)T, alanine (aspartate) aminotransferase; BCLC, stage, Barcelona-Clinic liver cancer stage; INR, international normalized ratio; PD-1, programmed cell death protein 1; Res, responders; Non-Res, non-responders.

### 3.2 Association of clinical responses with BA metabolism

**FIGURE 1:**
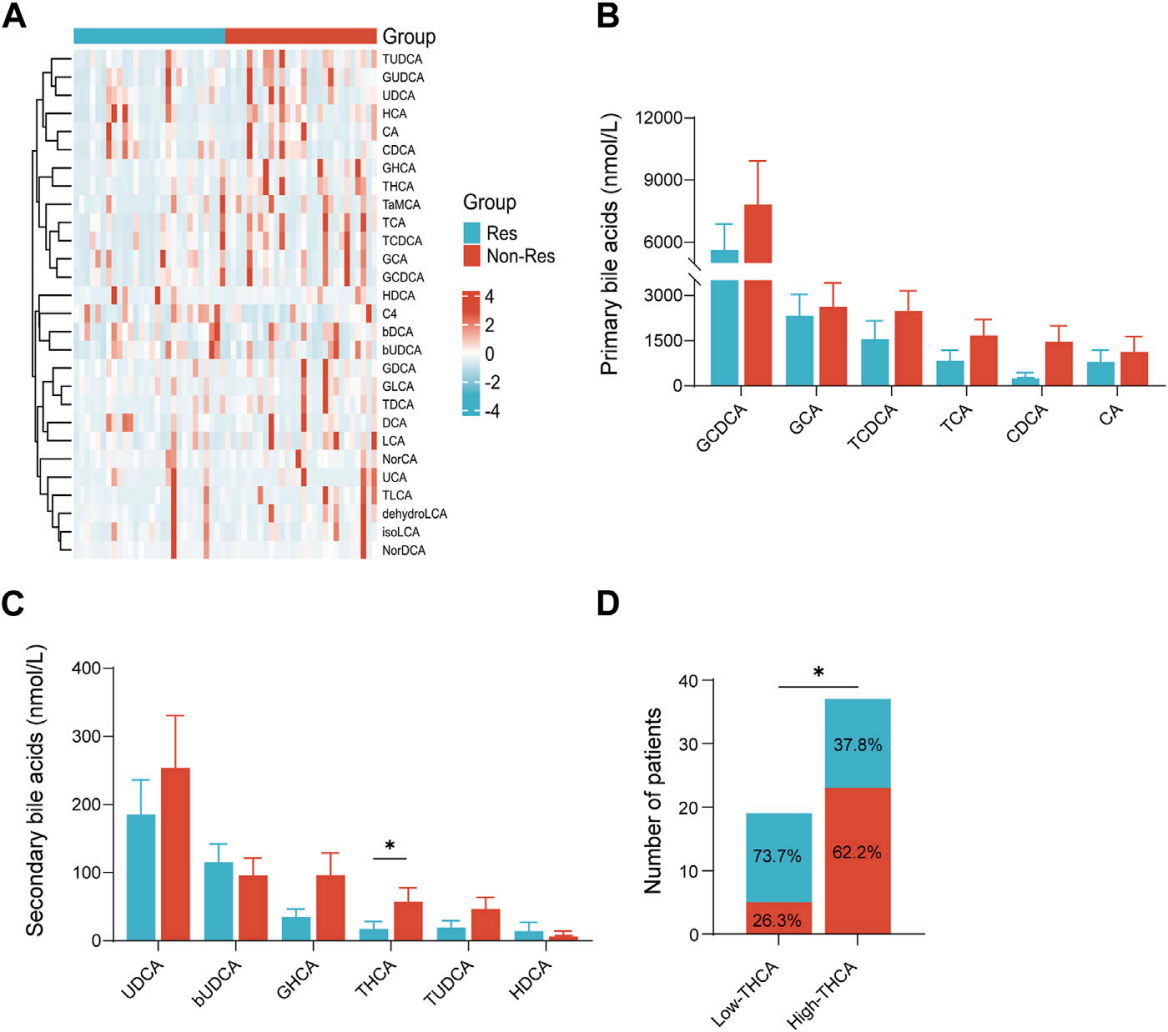
Plasma BA concentration in patients with hepatocellular carcinoma treated with tyrosine kinase inhibitors combined with programmed cell death-1 inhibitors therapy. **(A)** Heatmap of serum BA profiles in the Res and Non-Res groups; **(B)** primary BA levels in the Res and Non-Res groups; **(C)** secondary BA levels in the Res and Non-Res groups; and **(D)** The number of Res and Non-Res patients in the low and high THCA groups. *p < 0.05. BA, bile acid; Res, responders; Non-Res, non-responders; THCA, taurohyocholic acid.

The baseline plasma BA profiles in patients were examined using mass spectrometry to evaluate the levels and compositional features of BAs. The results of the BA measurements are shown in Figure 1. There were variations in BA composition between the two groups, and trends were observed in the heat map (Figure 1A). Although no significant differences were observed in the absolute concentrations of primary BAs between the two groups, the levels of primary BAs were generally higher in the Non-Res group than in the Res group (Figure 1B). Notably, among the secondary BAs, only taurohyocholic acid (THCA) levels were significantly reduced in the Res group compared with the Non-Res group (*p* < 0.05, Figure 1C).To investigate the correlation between BA metabolism and treatment response further, we categorized the 56 patients with HCC into high and low THCA groups. The optimal cut-off value for baseline serum THCA levels was determined to be 7.48 nmol/L by plotting the receiver operating characteristics (ROC) curve with the maximum Youden index. Our findings demonstrated that the response rate in the low THCA group was significantly better than that in the high THCA group. (73.7% vs. 37.8%, *p* < 0.05, Figure 1D). Taken together, these results suggest that baseline plasma BAs, especially THCA, correlate with the response to combination therapy in patients diagnosed with HCC, indicating that plasma BAs might be effective biomarkers for predicting the response to systematic drug treatment.

### 3.3 Changes in BAs during treatment

**FIGURE 2:**
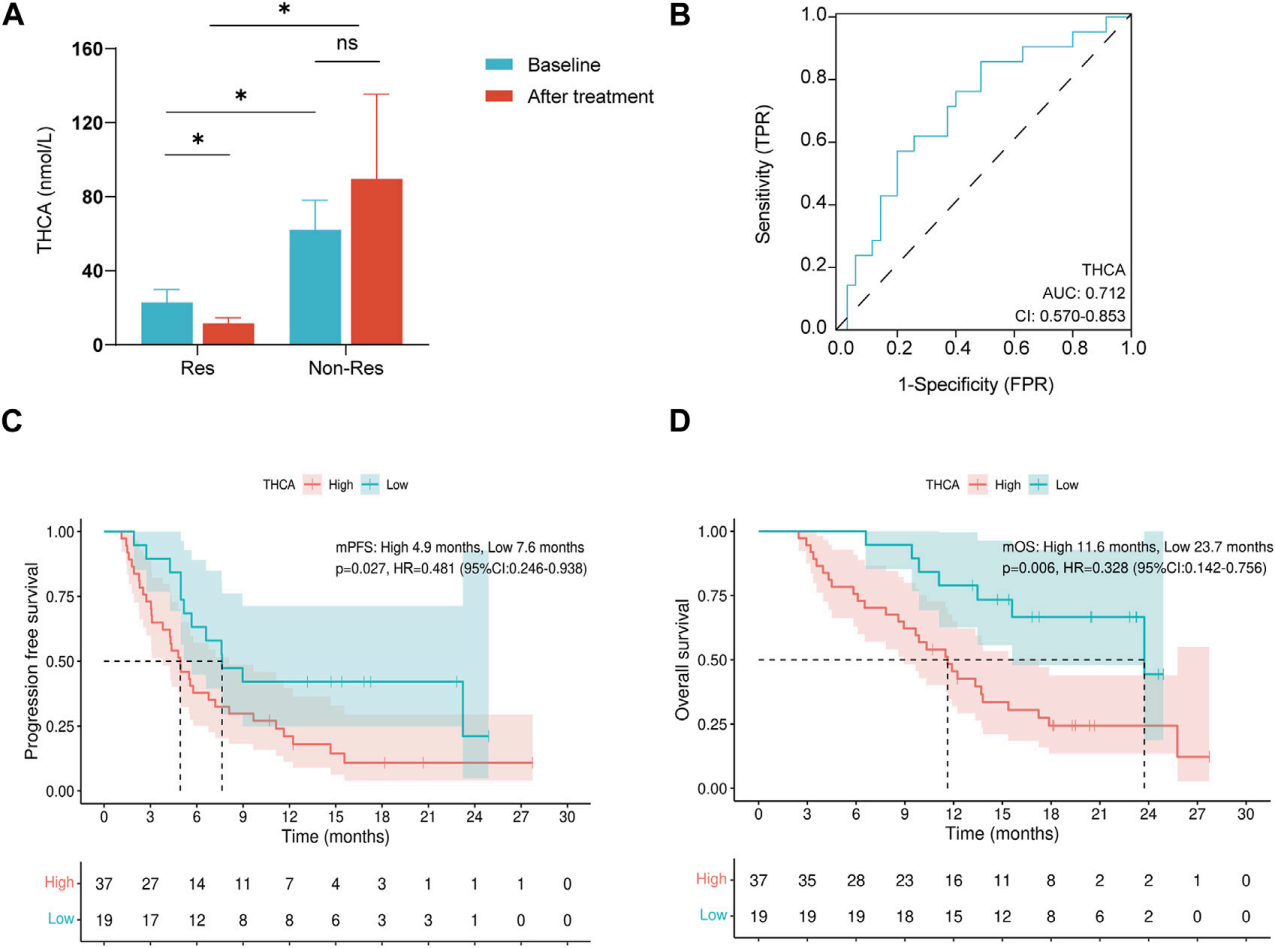
THCA is associated with tyrosine kinase inhibitors combined with programmed cell death-1 inhibitors therapy outcomes in patients with hepatocellular carcinoma. **(A)** Changes in plasma THCA levels at baseline and after treatment; **(B)** Receiver operating characteristic curve of THCA for overall survival in HCC; **(C)** Kaplan–Meyer curves for progression-free survival in patients with high and low baseline THCA levels; **(D)** Kaplan–Meyer curves for overall survival in patients with high and low baseline THCA levels. ns, not significant; *p < 0.05. THCA, taurohyocholic acid.

To understand the changes in BA metabolism during treatment, plasma THCA levels were detected at baseline and 3 months after treatment (three patients in the Res group were excluded from this analysis owing to a lack of 3-month post-treatment plasma samples). Compared to baseline plasma BA profiles, the Res group showed a significant decrease in THCA levels after treatment (*p* < 0.05, Figure 2A), whereas no significant change was observed in the Non-Res group. Furthermore, the Res group exhibited lower post-treatment THCA levels than the Non-Res group (*p* < 0.05, Figure 2A).

### 3.4 Association of survival with BA metabolism

According to the ROC analysis, THCA was an acceptable predictor of survival (AUROC: 0.712, Figure 2B). Therefore, THCA levels were investigated in survival analyses. Patients with lower THCA levels experienced a longer median PFS than patients with higher THCA levels (7.6 months vs. 4.9 months, *p* = 0.027, Figure 2C). An OS benefit was also observed in patients with lower THCA levels (median OS: 23.7 months vs. 11.6 months, *p* = 0.006, Figure 2D). Child–Pugh class (hazard ratio [HR]: 2.079, 95% confidence interval [CI]: 1.077–4.014, *p* = 0.029) and THCA (HR: 2.084, 95% CI: 1.065–4.078, *p* = 0.032) were identified as independent prognostic factors for PFS in multivariate Cox regression analysis (Table 2). In the univariate analysis of OS, only a high THCA level (HR, 3.049; 95% CI, 1.322–7.032; *p* = 0.009) was independently associated with poor OS (Table 3). Therefore, BAs are linked to survival in patients receiving combination therapy. Taken together, our findings indicate that the low THCA level in plasma might enhance the clinical response and prolong the survival of combination therapy, which can predict the survival benefit for patients with HCC receiving TKIs combined with PD-1 inhibitors therapy.

**TABLE 2:** Univariate and multivariate analyses of prognostic factors for progression-free survival.

Characteristic		Univariate analysis		Multivariate analysis	
		Hazard ratio (95% CI)	*p*-value	Hazard ratio (95% CI)	*p*-Value
Age	<60 years	1.480 (0.782–2.801)	0.228		
Sex	Female	0.754 (0.296–1.921)	0.555		
Diabetes	Yes	1.125 (0.596–2.124)	0.716		
Hypertension	Yes	0.673 (0.364–1.242)	0.205		
BMI	<24	0.719 (0.396–1.305)	0.279		
Child–Pugh class	B	2.080 (1.079–4.013)	**0.029**	2.079 (1.077–4.014)	**0.029**
BCLC stage	B	0.399 (0.096–1.654)	0.205		
Tumor size	≥5 cm	1.259 (0.664–2.386)	0.481		
Tumor number	Multiple	0.549 (0.300–1.007)	0.053		
Portal vein invasion	Yes	1.540 (0.737–3.219)	0.251		
Extrahepatic metastasis	Yes	0.800 (0.437–1.464)	0.469		
AFP	≥400 ng/mL	0.983 (0.538–1.796)	0.955		
Portal hypertension	Yes	1.030 (0.562–1.889)	0.924		
Cirrhosis	Yes	1.018 (0.400–2.590)	0.970		
ALT	>40 U/L	1.226 (0.672–2.239)	0.506		
AST	>40 U/L	1.255 (0.665–2.368)	0.483		
Treatment modality	Lenvatinib + PD-1 inhibitors	1.210 (0.618–2.369)	0.579		
THCA	High level	2.079 (1.066–4.057)	**0.032**	2.084 (1.065–4.078)	**0.032**

Values with *p* < 0.05 in variable analysis in Table are shown in bold.

Abbreviations: CI, confidence interval; BMI, body mass index; BCLC, stage, Barcelona-Clinic liver cancer stage; AFP, alpha-fetoprotein; AL(S)T, alanine (aspartate) aminotransferase; PD-1, programmed cell death protein 1; THCA, taurohyocholic acid.

**TABLE 3:** Univariate analyses of prognostic factors for overall survival.

Characteristics		Univariate analysis	
		Hazard ratio (95% CI)	*p*-value
Age	<60 years	1.269 (0.619–2.602)	0.515
Sex	Female	1.326 (0.510–3.448)	0.562
Diabetes	Yes	1.032 (0.500–2.131)	0.931
Hypertension	Yes	0.783 (0.398–1.543)	0.480
BMI	<24	1.063 (0.536–2.110)	0.861
Child–Pugh class	B	1.677 (0.816–3.446)	0.160
BCLC stage	B	0.643 (0.154–2.693)	0.546
Tumor size	≥5 cm	1.725 (0.826–3.603)	0.147
Tumor number	Multiple	1.058 (0.545–2.056)	0.867
Portal vein invasion	Yes	1.222 (0.552–2.706)	0.621
Extrahepatic metastasis	Yes	0.810 (0.405–1.623)	0.553
AFP	≥400 ng/mL	1.250 (0.639–2.446)	0.514
Portal hypertension	Yes	1.063 (0.531–2.129)	0.862
Cirrhosis	Yes	1.086 (0.381–3.093)	0.877
ALT	>40 U/L	1.281 (0.645–2.543)	0.479
AST	>40 U/L	1.753 (0.839–3.662)	0.136
Treatment modality	Lenvatinib + PD-1 inhibitors	1.200 (0.571–2.522)	0.631
THCA	High level	3.049 (1.322–7.032)	**0.009**

Values with *p* < 0.05 in variable analysis in Table are shown in bold.

Abbreviations: CI, confidence interval; BMI, body mass index; BCLC, stage, Barcelona-Clinic liver cancer stage; AFP, alpha-fetoprotein; AL(S)T, alanine (aspartate) aminotransferase; PD-1, programmed cell death protein 1; THCA, taurohyocholic acid.

### 3.5 Subgroup survival analyses

**FIGURE 3:**
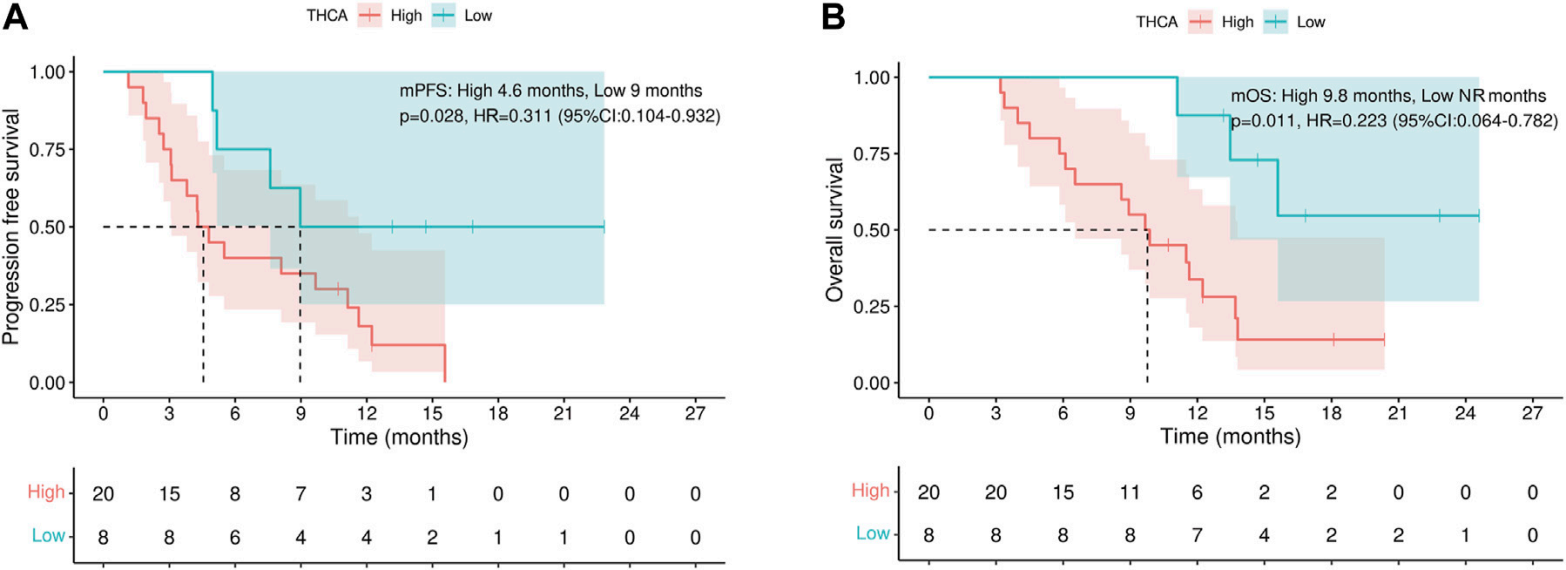
Progression-free and overall survival subgroup analysis. **(A)** THCA levels are associated with progression-free survival and **(B)** overall survival. NR, not reached; THCA, taurohyocholic acid.

To validate the above results, we repeated the survival analyses in the patient subgroup that received lenvatinib combined with sintilimab, the largest subgroup in our cohort. Of the 28 patients in the subgroup cohort, those with low THCA levels experienced a significantly prolonged PFS than patients with high THCA levels (median PFS: 9.0 months vs. 4.6 months, *p* = 0.028, Figure 3A). Importantly, patients with low THCA levels also experienced a significantly prolonged OS compared to those with high THCA levels (median OS: not reached [NR] vs. 9.8 months, *p* = 0.011, Figure 3B).

## 4 Discussion

To the best of our knowledge, this is the first study to link plasma BA metabolism with the treatment response to TKIs combined with PD-1 inhibitors treatment and survival in patients with HCC. Using plasma BA metabolic profile analysis, we demonstrated that plasma BA concentrations differed between the combination therapy Res and Non-Res groups of patients with HCC. Further analysis identified THCA as an independent predictor of survival. THCA levels were negatively associated with combination therapy response and survival in patients with HCC.

HCC is a solid tumor characterized by high heterogeneity, with varying immune microenvironments depending on the underlying liver disease. Immunotherapy combined with targeted therapy has proven more effective for virus-associated HCC (Qin et al., 2023). Immune checkpoint inhibitors contribute to antiviral therapy while exerting their anti-tumor mechanism of action. This may explain why the Chinese subgroups of the LEAP-002 and CARES-310 studies achieved a longer median overall survival. Patients with HBV-associated HCC in China bear a significant disease burden. Our study exclusively included patients with HBV-associated HCC, effectively mitigating disease background as a confounding factor. Further clinical studies are warranted to ascertain whether our current findings are applicable to hepatocellular carcinoma caused by other factors, such as HCV-associated, alcoholic, and non-alcoholic steatosis.

In addition to participating in the normal cholesterol metabolism pathway, BAs have been demonstrated to have a crucial role in the development of tumors and HCC (Sun et al., 2016; Wang et al., 2016). For instance, elevated levels of glycine- and taurine-conjugated primary bile acids have been linked to a heightened likelihood of chronic hepatitis B/C-related hepatocellular carcinoma (Petrick et al., 2020). Among patients undergoing anti-PD-1 immunotherapy for hepatobiliary tumors, those with elevated BA levels exhibited poorer treatment responses and prognosis (Mao et al., 2021). In the current study, the Res group exhibited lower levels of total bile acid (TBA) than the Non-Res group. In addition, a lower treatment response rate was observed in patients with high TBA levels (36.4% vs. 63.3%, Supplementary Figure S1A). However, these differences were not statistically significant, presumably owing to the relatively small cohort sample size. Further analysis of TBA levels before and after treatment revealed that TBA levels in the Res group were significantly decreased (*p* < 0.05, Supplementary Figure S1B). These observations suggest a close relationship between BAs and the response to immunotherapy. There were no significant differences between the two groups in the absolute concentrations of BAs, including chenodeoxycholic acid (CDCA), cholic acid (CA), and UDCA, which have been the focus of previous research (Figures 1A, B) (Ding et al., 2015; Liu et al., 2018; Režen et al., 2022). Another study reported that fecal UDCA concentrations were notably elevated in ICI responders compared to non-responders, whereas fecal lithocholic acid (LCA) concentrations were higher in patients with PD (Lee et al., 2022). This seems to disagree with our findings, in which an upward trend in plasma UDCA levels was observed in the Non-Res group. These conflicting results may be related to the different metabolic environments of plasma and feces; for example, the effects of intestinal flora and permeability (Mao et al., 2021; Shi et al., 2023). Previous studies have also indicated that the proportion of BAs in plasma and feces differ (Jia et al., 2020; Kasai et al., 2022). Recent studies have highlighted the crucial roles played by BAs in the development and function of T cells (Ding et al., 2022). The transformation of 3-oxoLCA into isoalloLCA by gut *Bacteroidetes* enhances naïve T cell differentiation into regulatory T cells, thus maintaining immune homeostasis (Hang et al., 2019; Song et al., 2020; Li et al., 2021).

Similar observations have been noted amongst patients with melanoma wherein elevated levels of circulating myeloid-derived suppressor cells (MDSCs) have been reported in non-responders to immunotherapy (Meyer et al., 2014; Tobin et al., 2017). This increase in the number of MDSCs may be due to taurodeoxycholate, which may also affect the immune-regulatory functions of these cells (Chang et al., 2018). These findings imply that patients with tumors experience diverse changes in BA concentration and/or composition, which could potentially regulate their response to antitumor therapies through mechanisms related to immune cells and intestinal flora.

Our study found that, among the BAs examined, THCA showed the most distinct differentiation between patients with HCC who responded to combination treatment and those who did not. Taurocholic acid (TCA) levels were also higher in the Non-Res group compared to the Res group. However, this difference did not reach statistical significance (Figure 1B), which could be due to the limited sample size of our study. In addition, THCA can be transformed into TCA. High levels of plasma TCA promote HCC development by inducing M2-like macrophage polarization and generating an immunosuppressive tumor microenvironment (Sun et al., 2022). Therefore, the interconversion of primary and secondary BAs has the potential to induce changes in immune cells within the tumor microenvironment, which may subsequently affect the treatment response. Further research is required to fully comprehend the mechanisms behind this association and explore potential therapeutic strategies targeting BA metabolism to improve treatment responses.

Multivariate Cox regression analysis demonstrated that the Child–Pugh class was an autonomous predictor of PFS, which may be related to changes in BAs. Liver dysfunction can impact BA metabolism in numerous ways, including hampered hepatic BA clearance, heightened BA synthesis, and alterations in the gut microbiome. These changes may cause modifications to the production of secondary and tertiary BAs (Fuchs and Trauner, 2022). Conversely, the dysregulation of BA metabolism may lead to impaired liver function (Ahmad and Haeusler, 2019). Alterations in liver function can affect the response to systemic therapies. Therefore, it is crucial to consider liver function and BA metabolism when evaluating the efficacy and outcomes of anti-tumor therapies in patients with HCC.

Furthermore, we assessed whether variations in the impact of distinct bile acids on patients’ overall and progression-free survival existed. Subsequent analysis revealed that the concentration of certain bile acids, including C4, TLCA, and TUDCA, significantly affected patients’ PFS and/or OS despite no differences in baseline levels (Supplementary Tables S1, S2). The small sample size may account for this, and the findings in this study segment require validation in a larger patient cohort.

Our study has some limitations that must be considered. First, this was a retrospective, single-center study with a limited sample size. This may have resulted in a degree of information and selection bias. Therefore, prospective studies with larger clinical cohorts are required to validate our findings and hypotheses. Second, the treatment regimen for this study cohort was relatively complex. Therefore, to strengthen the hypothesis of our study, we conducted a subgroup analysis specifically focusing on the treatment regimen of lenvatinib plus sintilimab, and we will explore subgroup analyses of other combination regimens in the future with an expanded sample size. Our study lacked fecal metagenomic and metabolomic information, which would aid the understanding of the relationship between the gut microbiota and host metabolism. Finally, we investigated the potential reasons for the lack of response to systemic therapy by examining plasma BA metabolism. However, the specific mechanisms underlying this lack of treatment response require further investigation, both *in vivo* and *in vitro*.

In conclusion, we have demonstrated the significant role of BAs in the therapeutic outcomes of HCC. In addition, we suggest, for the first time, that peripheral blood THCA might be a valid biomarker, the levels of which independently predict TKIs combined with PD-1 inhibitors therapy response in patients with HCC. These findings highlight a promising therapeutic approach to enhance the treatment efficacy of systemic anti-tumor agents through the modification of BA metabolites. More research is needed to clarify the role of BAs in the treatment of HCC completely. However, by considering the role of BAs in the treatment response, we may be able to develop personalized treatment approaches that consider individual variations in BA metabolism, ultimately leading to improved outcomes in patients with HCC.

## Figures and Tables

**Table:** 

**6-KetoLCA**	6-ketolithocholic acid
**AFP**	alpha-fetoprotein
**AL(S)T**	alanine(aspartate) aminotransferase
**AUROC**	area under the receiver operating characteristic curve
**BA**	bile acid
**BCLC stage**	Barcelona-Clinic liver cancer stage
**bDCA**	b-deoxycholic acid
**BMI**	body mass index
**bUDCA**	b-ursodeoxycholic acid
**CA**	cholic acid
**CDCA**	chenodeoxycholic acid
**CI**	confidence interval
**CR**	complete response
**C4**	7a-Hydroxy-cholestene-3-one
**DCA**	deoxycholic acid
**GCA**	glycocholic acid
**GCDCA**	chenodeoxycholic acid glycine conjugate
**GDCA**	deoxycholic acid glycine conjugate
**GHCA**	glycohyocholic acid
**GLCA**	lithocholic acid glycine conjugate
**GUDCA**	glycoursodeoxycholic acid
**HCA**	hyocholic acid
**HCC**	hepatocellular carcinoma
**HDCA**	Hyodeoxycholic acid
**HR**	hazard ratio
**ICI**	immune checkpoint inhibitor
**INR**	international normalized ratio
**isoLCA**	isolithocholic acid
**LCA**	lithocholic acid
**LCA.3S**	lithocholic acid 3-sulfate
**MDSCs**	myeloid-derived suppressor cells
**OS**	overall survival
**PR**	partial response
**PD-1**	programmed cell death protein 1
**PFS**	progression-free survival
**PD**	progressive disease
**RECIST**	Response Evaluation Criteria in Solid Tumors
**ROC**	receiver operating characteristic curve
**SD**	stable disease
**TaMCA**	Tauro-α-muricholic acid
**TBA**	total bile acid
**TCA**	taurocholic acid
**TCDCA**	taurochenodesoxycholic acid
**TDCA**	Taurodeoxycholic acid
**THCA**	taurohyocholic acid
**TLCA**	Taurolithocholic acid
**TUDCA**	tauroursodeoxycholic acid
**UCA**	ursocholicacid
**UDCA**	ursodeoxycholic acid
**NorCA**	Norcholic acid.

## Data Availability

The original contributions presented in the study are included in the article/Supplementary Material, further inquiries can be directed to the corresponding authors.
