# Supplementary material for: Taurohyocholic acid acts as a potential predictor of the efficacy of tyrosine kinase inhibitors combined with programmed cell death-1 inhibitors in hepatocellular carcinoma
Source: Front Pharmacol. 2024 Feb 23;15:1364924. doi: 10.3389/fphar.2024.1364924 (PMC10920247; doi:10.3389/fphar.2024.1364924)
Supplement: Supplementary file 1 [file Presentation1.PDF]

Supplementary Material

1 Supplementary Figures and Tables

1.1 Supplementary Figures

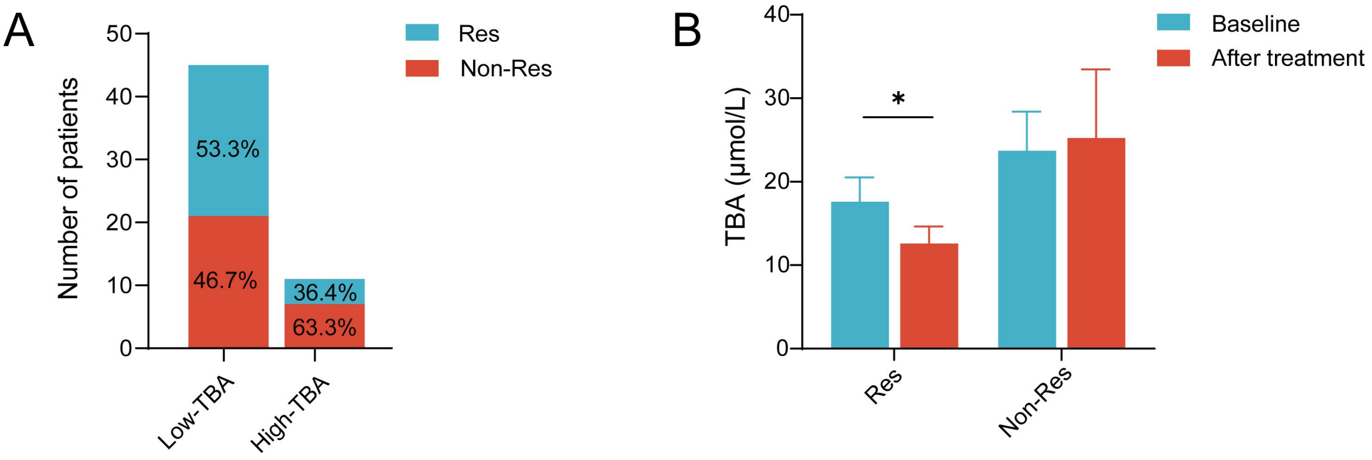

Supplementary Figure 1. TBA concentrations in hepatocellular carcinoma patients treated with tyrosine kinase inhibitors plus programmed cell death-1 inhibitors therapy. (a) Number of Res and Non-Res patients in the low and high TBA groups. (b) Changes in plasma TBA levels at baseline and after treatment. *p<0.05. TBA, total bile acid; Res, responders; Non-Res, non-responders.

1.2 Supplementary Tables

Supplementary Table 1. The correlation between the expression levels of various bile acids and the progression-free survival of patients was examined

Bile acid	Cut-off	Hazard ratio (95% CI)	P value	Correlation
6-KetoLCA	2.86	0.501(0.274-0.918)	0.022	Negative
C4	597	2.55 (1.124-5.768)	0.002	Positive
CA	678.03	0.442 (0.194-1.004)	0.046	Negative
GLCA	46.01	0.441(0.214-0.909)	0.022	Negative
HCA	76.74	0.466(0.252-0.862)	0.012	Negative
HDCA	6.48	0.417(0.227-0.768)	0.004	Negative
LCA.3S	10.98	0.436(0.233-0.813)	0.007	Negative

THCA	7.48	0.481 (0.2246-0.938)	0.027	Negative
TLCA	6.99	0.43 (0.209-0.885)	0.019	Negative
TUDCA	68.14	0.353 (0.166-0.749)	0.005	Negative

Abbreviations: CI, confidence interval; 6-KetoLCA, 6-ketolithocholic acid; C4, 7a-Hydroxy-cholestene-3-one; CA, cholic acid; GLCA, lithocholic acid glycine conjugate; HCA, hyocholic acid; HDCA, Hyodeoxycholic acid; LCA.3S, lithocholic acid 3-sulfate; THCA, taurohyocholic acid; TLCA, Taurolithocholic acid; TUDCA, tauroursodeoxycholic acid.

Supplementary Table 2. The correlation between the expression levels of various bile acids and the overall survival of patients was examined

Bile acid	Cut-off	Hazard ratio (95% CI)	P value	Correlation
6-KetoLCA	2.86	0.493(0.252-0.968)	0.036	Negative
C4	597	4.54(1.383-14.887)	0.006	Positive
CA	678.03	0.346 (0.141-0.849)	0.015	Negative
GHCA	33.59	0.392(0.192-0.801)	0.008	Negative
GLCA	46.01	0.409(0.181-0.920)	0.026	Negative
GUDCA	1472.8	0.344(0.131-0.907)	0.024	Negative
HCA	76.74	0.344(0.171-0.691)	0.002	Negative
LCA	42	0.417(0.195-0.888)	0.019	Negative
NorCA	12.57	0.309(0.153-0.624)	<0.001	Negative
TaMCA	13.86	0.393 (0.182-0.848)	0.014	Negative
TCA	388.35	0.405 (0.203-0.809)	0.008	Negative
TCDCA	652.49	0.448 (0.218-0.922)	0.025	Negative
THCA	7.48	0.328 (0.142-0.756)	0.006	Negative
TLCA	6.99	0.341 (0.156-0.744)	0.005	Negative
TUDCA	68.14	0.207 (0.092-0.466)	<0.001	Negative
UDCA	614.94	0.375 (0.142-0.991)	0.004	Negative

Abbreviations: CI, confidence interval; 6-KetoLCA, 6-ketolithocholic acid; C4, 7a-Hydroxy-cholestene-3-one; CA, cholic acid; GHCA, glycohyocholic acid; GLCA, lithocholic acid glycine conjugate; GUDCA, glycoursodeoxycholic acid; HCA, hyocholic acid; LCA, lithocholic acid; NorCA, Norcholic acid; TaMCA, Tauro-α-muricholic acid; TCA, taurocholic acid; TCDCA, taurochenodesoxycholic acid; THCA, taurohyocholic acid; TLCA, Taurolithocholic acid; TUDCA, tauroursodeoxycholic acid; UDCA, ursodeoxycholic acid.
